# Bridging a gap in iron-sulfur cluster assembly

**DOI:** 10.7554/eLife.10479

**Published:** 2015-09-09

**Authors:** Erin L. McCarthy, Squire J. Booker

**Affiliations:** Department of Biochemistry and Molecular Biology, Pennsylvania State University, University Park, United States; Departments of Biochemistry and Molecular Biology and of Chemistry, Pennsylvania State University, University Park, United Statessjb14@psu.edu

**Keywords:** post-translational modification, metal biology, iron-sulfur protein biogenesis, mitochondria, CIA machinery, human, *S. cerevisiae*

## Abstract

The cellular machinery that incorporates iron-sulfur clusters into proteins is directed to particular targets by adaptor proteins.

**Related research article** Paul VD, Mühlenhoff U, Stümpfig M, Seebacher J, Kugler KG, Renicke C, Taxis C, Gavin AC, Pierik AJ, Lill R. 2015. The deca-GX_3_ proteins Yae1-Lto1 function as adaptors recruiting the ABC protein Rli1 for iron-sulfur cluster insertion. *eLife* **4**:e08231. doi: 10.7554/eLife.08231

**Image** A complex network of proteins is required to assemble the clusters of iron and sulfur ions that some proteins need to work

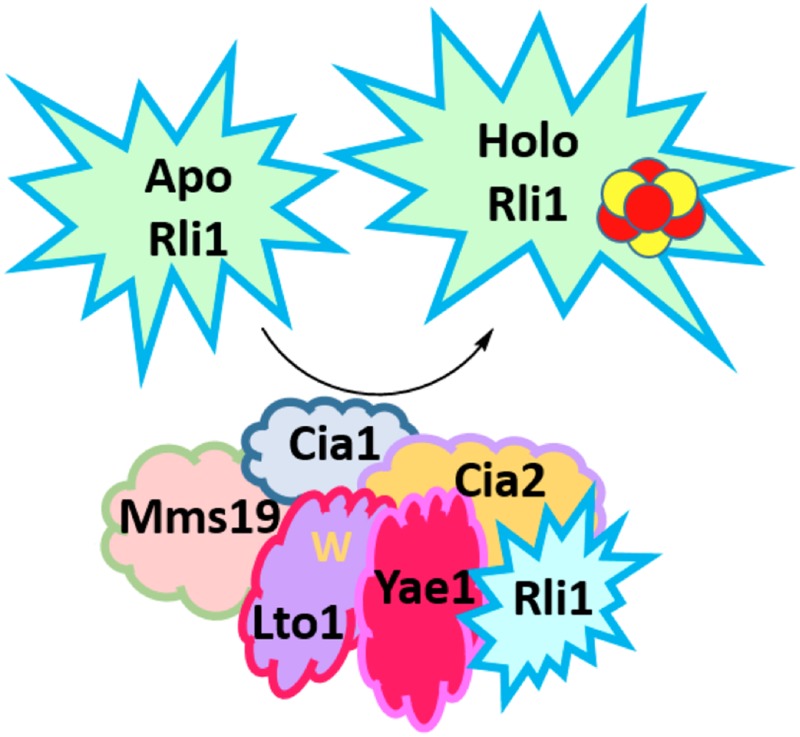

Many proteins must tightly bind to other molecules – known as prosthetic groups – in order to carry out their roles. Iron-sulfur (or Fe-S) clusters are among the most ancient and versatile prosthetic groups in nature. They are also essential for many important biological tasks, including enzyme catalysis and gene regulation (Sharma et al., 2010). Consistent with their primordial origin, Fe-S clusters can readily assemble onto their protein targets without any help in the presence of iron and sulfide. This process, however, is too slow and uncontrolled for the metabolic needs of the cell. Indeed, despite their simple chemical makeup, making Fe-S clusters in living organisms is a complex undertaking that involves a highly coordinated network of proteins setting specific events in motion at designated times. Identifying these proteins and their roles is a major goal of research in this area (Maio and Rouault, 2015; Paul and Lill, 2015). Now, in eLife, Roland Lill of the Philipps-Universität Marburg and co-workers in Germany – including Viktoria Désirée Paul as first author – identify two proteins that direct a key part of the cellular machinery that makes iron-sulfur clusters to a particular target protein (Paul et al., 2015).

The core process of Fe-S cluster biosynthesis involves three steps. The cluster is first assembled onto an intermediate scaffold protein. The recipient protein is then recruited to the scaffold complex, usually with the aid of other proteins, and the newly assembled cluster is subsequently transferred to the recipient protein (Figure 1). In humans, problems in any one of these steps can result in potentially fatal diseases. For example, Friedreich's ataxia is a neurodegenerative disease in which a deficiency in the Fe-S cluster assembly protein, frataxin, causes toxic levels of iron to accumulate (Maio and Rouault, 2015).

**Figure 1.**
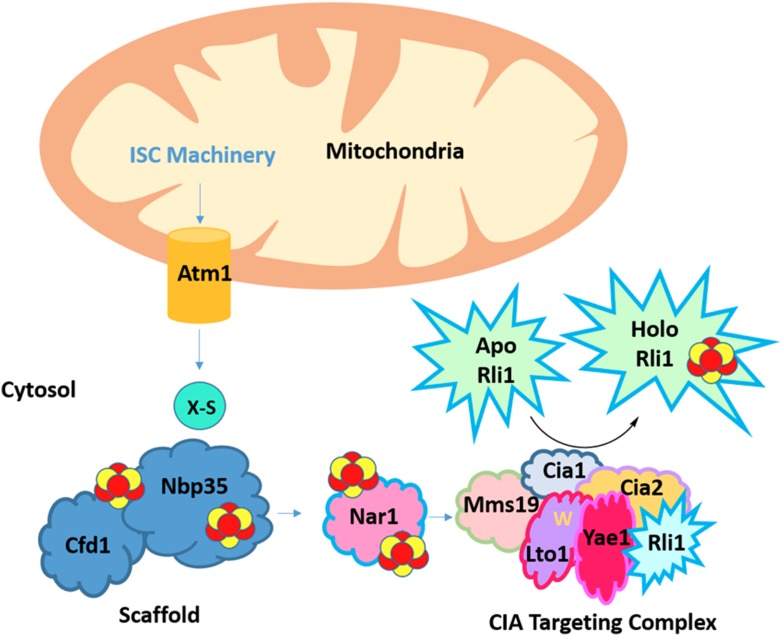
A complex network of proteins asssembles and transfers an iron-sulfur cluster to the essential protein Rli1. The iron-sulfur cluster (ISC) machinery, located in mitochondria, exports an unknown sulfur-containing compound (X-S) via the Atm1 transporter to the cytosol. Using this sulfur atom and an undefined source of iron, a 4Fe-4S cluster that contains four iron atoms and four sulfur atoms (show as red and yellow circles) is assembled on a scaffold complex consisting of the proteins Cfd1 and Nbp35. The assembled cluster is transferred to Nar1 and the CIA targeting complex (Mms19, Cia1, and Cia2) for subsequent insertion into target proteins. This changes the target from its ‘apo’ (without prosthetic group) form to a ‘holo’ form (with prosthetic group). Paul et al. found that cluster assembly on Rli1 requires the CIA targeting complex and two Rli1-specific adaptor proteins, Yae1 and Lto1. In a unique binding mechanism, a conserved tryptophan residue (shown as a yellow ‘W’) in the Lto1 protein interacts with the CIA targeting complex, while Yae1 recruits Rli1. In addition, conserved deca-GX_3_ motifs in Yae1 and Lto1 are required to form the CIA targeting complex.

Eukaryotic cells contain several membrane-enclosed structures – including the nucleus, and the mitochondria that generate energy for the cell – that are surrounded by a fluid called the cytosol. Two systems exist in eukaryotes for creating Fe-S clusters. The first, the iron-sulfur cluster (ISC) system, is localized in the mitochondria. The second, known as the cytosolic iron-sulfur cluster assembly (CIA) system, exists in the cytosol and is responsible for adding Fe-S clusters to proteins made in the cytosol or nucleus. The CIA system also depends on the iron-sulfur cluster system to generate an elusive sulfur-containing compound that it needs to work (Kispal et al*.*, 1999; Paul and Lill, 2015; Figure 1).

Given that Fe-S proteins have a diverse range of structures and roles, one of the major questions in the field of Fe-S cluster biosynthesis is: how does each of the systems recognize their protein targets? To shed light on this issue in the CIA system, Paul et al. focused on how Fe-S clusters are incorporated into an essential cytosolic Fe-S protein called Rli1, which is a known target of the CIA machinery. Using a clever biochemical approach to identify proteins that bind to targets of interest, Paul et al. pinpointed two proteins – called Yae1 and Lto1 – that interact strongly with the CIA machinery. Previous work suggested that these proteins also interact with Rli1 (Krogan et al*.*, 2006).

Using a radioactive isotope of iron to monitor how Fe-S clusters form in yeast cells, Paul et al. further probed how Yae1 and Lto1 help to assemble iron-sulfur proteins. Under conditions in which Yae1 and Lto1 were absent or greatly diminished, little iron was incorporated into Rli1, signaling the absence of Fe-S clusters on the protein. Further experiments showed that both proteins are necessary for Fe-S clusters to be incorporated into Rli1. By contrast, depleting Yae1 and Lto1 had no effect on the formation of clusters on three other nuclear Fe-S target proteins. Together, these studies established that both Lto1 and Yae1 bind to the CIA machinery and specifically recruit Rli1 to the CIA targeting complex, where Fe-S clusters are incorporated into Rli1.

The sequence of amino acids that makes up a protein affects how the protein works, and short sequences that are particularly important for the activity of the protein are known as motifs. Both Yae1 and Lto1 contain a motif that is rare in nature, called deca-GX_3_. Paul et al. substituted various glycine molecules in this motif in Lto1 with other amino acids to assess whether the motif is important for the protein–protein interactions that recruit Rli1 to the CIA machinery. Further experiments investigated the effect of replacing a highly conserved tryptophan amino acid that is found at the C-terminal end of Lto1. These experiments revealed that Lto1 needs its C-terminal tryptophan in order to bind to the CIA machinery, and that the deca-GX_3_ motif is important for Lto1 to bind to both the CIA machinery and Yae1. In turn, Yae1 mediates binding to Rli1 (Zhai et al., 2014). Co-expressing the human equivalents of Yae1 and Lto1 in yeast cells that lack these proteins restores the ability of the cells to add Fe-S clusters to Rli1. This complementation reaffirms that these proteins have remained more or less unchanged throughout evolution.

Paul et al.'s findings bridge an important gap in our understanding of how Fe-S clusters are made in the cytosol of cells. They show that specific adaptor proteins can link the Fe-S cluster biosynthetic machinery to specific target proteins. In their model, the adaptor protein Yae1 binds to Rli1 and then recruits it to the CIA machinery via interactions that it makes with the deca-GX_3_ motif on Lto1. In turn, Lto1 requires a tryptophan amino acid in its C-terminal region to bind to the CIA machinery. Significant progress has been made in the field, yet further *in vivo* studies are necessary for understanding Fe-S cluster assembly, and ultimately, how the dysfunction of this process results in disease. Despite several hypotheses, the source of the iron needed for cluster assembly and the mode by which it is delivered to the scaffold protein is unknown. It is also not well understood why the sulfur that is needed for the CIA machinery needs to be exported from the mitochondria, and the identity of this sulfur compound remains to be determined. Importantly, the paradigm established by Lill et al. in this work could extend to other Fe-S proteins that also use protein-specific adaptor proteins for their cluster assembly.
